# Differences in HIV Markers between Infected Individuals Treated with Different ART Regimens: Implications for the Persistence of Viral Reservoirs

**DOI:** 10.3390/v12050489

**Published:** 2020-04-27

**Authors:** Gilles Darcis, Ben Berkhout, Alexander O. Pasternak

**Affiliations:** 1 Infectious Diseases Department, Liège University Hospital, 4000 Liège, Belgium; gdarcis@chuliege.be; 2 Laboratory of Experimental Virology, Department of Medical Microbiology, Amsterdam UMC, University of Amsterdam, 1105 AZ Amsterdam, The Netherlands; b.berkhout@amsterdamumc.nl

**Keywords:** HIV, latent reservoirs, residual viremia, antiretroviral therapy

## Abstract

In adherent individuals, antiretroviral therapy (ART) suppresses HIV replication, restores immune function, and prevents the development of AIDS. However, ART is not curative and has to be followed lifelong. Persistence of viral reservoirs forms the major obstacle to an HIV cure. HIV latent reservoirs persist primarily by cell longevity and proliferation, but replenishment by residual virus replication despite ART has been proposed as another potential mechanism of HIV persistence. It is a matter of debate whether different ART regimens are equally potent in suppressing HIV replication. Here, we summarized the current knowledge on the role of ART regimens in HIV persistence, focusing on differences in residual plasma viremia and other virological markers of the HIV reservoir between infected individuals treated with combination ART composed of different antiretroviral drug classes.

## 1. Introduction

Antiretroviral therapy (ART) represents an exceptional achievement of modern medicine. ART drastically decreases morbidity and mortality of human immunodeficiency virus (HIV)-infected individuals and radically reduces the risk of virus transmission [1]. During the last decade, single-tablet regimens have significantly advanced HIV management by reducing the pill burden and improving adherence to therapy [2]. However, ART is not curative and must be continued for life. The major hurdle to virus eradication resides in the persistence of latent HIV proviruses that form the viral reservoirs [3,4]. Several potential mechanisms of HIV reservoir persistence have been proposed. This review summarized the current knowledge about the role of different ART regimens in HIV persistence.

## 2. Persistence of HIV Reservoirs on ART

There is so far no consensus definition of latent HIV reservoirs. Historically, the prevailing view of HIV latency has been that it is mostly regulated at the transcriptional level [5]. However, virus latency does not require a complete shutdown of viral gene expression, but only the absence of infectious progeny production [6]. In fact, numerous studies demonstrated that some persistent HIV proviruses could produce viral RNA and even proteins, highlighting that transcriptional and post-transcriptional mechanisms act in concert to repress HIV expression [7,8,9,10,11,12,13,14]. HIV latency has thus to be perceived as a dynamic continuum [15,16], with the possibility for some latent proviruses to be the source of viral RNA and proteins.

Another issue is whether all proviruses should be included in the definition of the HIV reservoir or whether this should be restricted to the very small (<10%) replication-competent fraction of the proviral load. Indeed, the absolute majority of the integrated proviruses are genetically defective [17,18,19,20]. As these defective proviruses are incapable of triggering viral rebound following ART interruption, it has been suggested to restrict the definition of the HIV reservoir to the “replication-competent reservoir”, a small number of intact proviruses that should be eliminated in order to avoid viral resurgence after therapy interruption [21]. This definition ignores >90% of all proviruses that are replication-defective, but can nevertheless produce viral (or novel) RNA species, proteins, or even defective virus particles, potentially contributing to chronic immune activation and inflammation despite ART [22,23,24,25,26,27,28,29]. The definition of the HIV reservoir thus could be extended to include defective proviruses, as long as they contribute to residual HIV pathogenesis under ART – something that is yet to be shown.

The HIV reservoir is established early during primary HIV infection [30,31]. However, in the untreated infection, the reservoir seems to turn over rather quickly, and most proviral DNA sequences in peripheral blood mononuclear cells from ART-treated individuals were recently shown to match circulating HIV variants detected shortly before the start of therapy [32,33,34,35]. In most subjects, the HIV DNA load decreases during the first year after ART initiation, but the decay slows down during years 1–4, and the HIV DNA load eventually reaches a plateau [36]. Moreover, the HIV proviral load increases over time in a significant proportion of individuals [36,37]. This contrasts with the rapid decay of intact proviruses on ART [19,20] and implies possible preferential clonal expansion of cells harboring defective proviruses, although this view is still open to debate.

The complexity and dynamic nature of the viral reservoirs are constantly being revisited. HIV latency is established in several cell types, including various T-cell subsets that are characterized by distinct phenotypes and metabolic properties, but also non-T cells from the myeloid lineage [38,39,40]. Most of the HIV latent reservoirs reside in various tissues, such as the lymph nodes, the central nervous system, the gut-associated lymphoid tissue, the testes, and the urethra [41,42,43,44,45]. A better understanding of these heterogeneous reservoirs and their evolution during long-term ART is an indispensable prerequisite to the design of strategies aimed at eradicating HIV, and more precise identification of the latently infected cells would facilitate studies of the HIV reservoirs [46]. 

## 3. Sources of HIV Residual Viremia on ART 

In most cases, HIV-infected individuals achieve virological suppression in plasma to below the limit of quantification of commercial assays within a couple of months after starting ART. The word “undetectable” is often improperly used to refer to the viral load (VL) below the limit of quantification. Indeed, a PCR signal can sometimes be detected by commercial assays below the limit of quantification [47]. Unquantifiable VLs can thus be categorized as undetectable or detectable. Using ultrasensitive RT-qPCR assays, most individuals with VLs below the limit of quantification of commercial assays still have detectable HIV RNA in plasma, averaging around 1–3 copies/mL [48,49,50,51,52,53,54]. A number of excellent reviews on persistent residual viremia (RV) in ART-treated individuals have been published [55,56,57,58,59,60,61,62,63], and a mathematical model of its origins has been put forward [64]. Importantly, RV can contribute to the viral rebound following ART interruption, at least in some cases [65].

The source of RV despite ART has been the subject of a longstanding debate in the field, likely because there may be multiple sources that may vary between infected individuals. In fact, this debate reflects the wider debate on the mechanisms of HIV persistence in ART-treated individuals. Just as it is generally accepted that HIV reservoir persists primarily by longevity and proliferation of cells infected prior to ART initiation [66,67,68], the most prevalent view on RV is that, in most individuals, it arises from reactivation of latently infected cells (either cells infected before ART initiation or the progeny of such cells) in the peripheral blood and tissues [55,69].

Another potential source of RV (and a potential mechanism of HIV persistence) is ongoing viral replication despite ART (reviewed in [70]), which can result from insufficient penetration of a number of antiretroviral drugs into tissues and anatomic sanctuaries [71,72,73,74,75,76,77], causing reduced local drug concentrations in tissue sites as compared to peripheral blood. Direct demonstration of infection of new cells in an individual on ART is extremely difficult if not impossible, but a number of studies attempted to indirectly prove the occurrence of residual HIV replication by demonstrating virus evolution on ART. Most studies could not detect virus evolution, including the emergence of novel drug-resistance mutations, in most individuals [78,79,80,81,82,83,84,85]. This lack of significant virus evolution on ART is considered one of the strongest arguments against residual HIV replication. While the absence of evidence is not evidence of absence and even deeper and more comprehensive studies into viral evolution in ART-treated individuals may be needed, the prevailing consensus is that no further HIV sequence diversification from pre-therapy occurs in blood or tissues of individuals on suppressive ART [86]. However, the debate on residual HIV replication under ART recently regained momentum with the publication of an intensive study of HIV evolution in lymphoid tissue that revealed a temporal structure of viral populations during the first 6 months of ART [73], although other investigators challenged that conclusion [32,87,88], e.g., because no long-term treated individuals were included. Finally, it must be noted that if residual replication occurs intermittently, with short infection chains constantly arising and terminating, then nucleotide substitutions are expected to be sporadic and not linked by temporal structure unless the individual is sampled very intensively [64,89]. This implies that failure to detect virus evolution does not per se form unequivocal proof that residual viral replication does not occur, although it does indicate that residual replication probably occurs only in a subset of ART-treated individuals and at a low level.

## 4. Impact of ART Intensification on Residual Viremia and Other HIV Reservoir Markers

**Figure 1:**
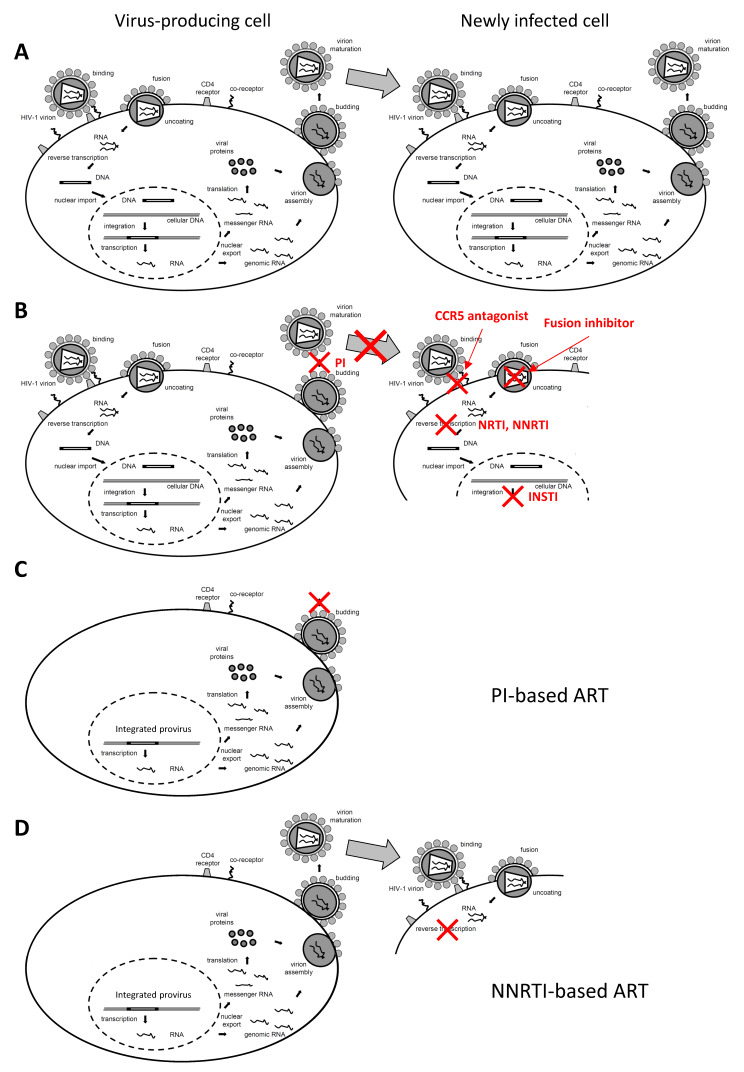
HIV replication cycle and the effects of antiretroviral drugs. (**A**) Untreated infection. After the virus particle attaches to receptors on the cell surface, the HIV RNA genome enters the cytoplasm and is reverse-transcribed into DNA, which is transported to the nucleus where it integrates into the host cell genome and serves as a template for viral transcription. Transcription of the proviral DNA template and alternative RNA splicing creates spliced and unspliced viral RNA species, encoding the viral structural and accessory proteins. All viral transcripts are exported into the cytoplasm, where translation and assembly and processing of the retroviral particle take place. The cycle is completed by the budding of retroviral particles from the cell and their maturation that produces infectious virions, which can infect new cells. (**B**) Different classes of antiretroviral drugs, such as protease inhibitors (PIs), CCR5 antagonists, fusion inhibitors, nucleoside and non-nucleoside reverse transcriptase inhibitors (NRTIs and NNRTIs), and integrase strand transfer inhibitors (INSTIs), prevent infection of new cells by blocking (depicted with red X) different stages of the HIV replication cycle. Although these regimens act at different stages of the viral replication cycle, neither of them inhibits HIV RNA transcription or virus production in long-lived reservoir cells that were infected prior to ART initiation or the progeny of such cells. However, the infection of new cells is prevented. Note that most reservoir cells in individuals on ART do not produce virus at any given time, but can be reactivated to do so in response to immune stimuli. Note also that PIs do not prevent virus production but block virion maturation, rendering virus particles noninfectious. In NNRTI- and INSTI-treated individuals, virus particles are infectious and can enter new cells, but the infection is blocked at the stage of reverse transcription or DNA integration, respectively. (**C**) PI-based ART. (**D**) NNRTI-based ART.

More than 20 different antiretroviral drugs belonging to six main classes are currently approved for clinical use [90]. Depending on the class, these drugs block different steps of the HIV replication cycle, such as reverse transcription, proviral integration, or virus particle maturation (Figure 1). All these drugs act by preventing the infection of new cells and are not expected to inhibit HIV RNA transcription or virus production in cells that were infected prior to ART initiation, including the long-lived reservoir cells. Current ART regimens typically consist of two nucleotide or nucleoside reverse transcriptase inhibitors (NRTI) and the third drug of another class (e.g., a non-nucleoside reverse transcriptase inhibitor (NNRTI), a protease inhibitor (PI), or an integrase strand transfer inhibitor (INSTI)), although dual therapies attained a significant position in the most recent guidelines (https://www.eacsociety.org/files/2019_guidelines-10.0_final.pdf; https://aidsinfo.nih.gov/guidelines/html/1/adult-and-adolescent-arv/11/what-to-start).

The possibility of ongoing HIV replication in sanctuaries, where drug penetration is suboptimal, and its potential contribution to the residual viremia roused a considerable interest in drug intensification: adding one or more antiretroviral drugs to boost the ART regimen [91,92,93,94,95,96,97,98,99,100]. Most intensification studies added an INSTI (raltegravir or dolutegravir) to the ART regimen. The impact of raltegravir intensification in individuals treated with ritonavir-boosted PI monotherapy has been documented by Puertas et al. [101]. Raltegravir intensification in this specific context resulted in a transient increase in HIV episomal DNA levels in a significant proportion of participants, along with decreases in the CD8+ T-cell activation levels. Episomal DNA (mostly detectable in the form of 2-long terminal repeat (2-LTR) circles) is a byproduct of HIV integration and has been proposed to mark recently infected cells, although this has been disputed [102,103,104,105,106,107]. In any case, the accumulation of episomal DNA observed upon blocking HIV integration by the addition of an INSTI would reveal ongoing integration events prior to intensification. Because all other antiretroviral drug classes act upstream of INSTIs (Figure 1B), this implies that complete rounds of HIV replication (infection of new cells) had been ongoing pre-intensification. Confirming this, raltegravir also decreased the proportion of individuals with intermediate levels of RV (10–60 HIV RNA copies/mL).

The impact of raltegravir or dolutegravir intensification on HIV persistence is much more controversial when applied to individuals treated with a three-drug regimen. On the one hand, several studies could not demonstrate any effect of intensification on RV [92,93,97]. On the other hand, Buzon et al. reported that raltegravir intensification of a three-drug suppressive ART regimen resulted in a specific and transient increase of the episomal DNA levels in a significant proportion of ART-treated individuals [94]. Furthermore, in subjects with these episomal DNAs, immune activation was higher at baseline and normalized after raltegravir intensification. Interestingly, these subjects were frequently treated with a PI-based ART regimen. The results of the Buzon study were initially disputed by other investigators [108,109,110], but subsequently, Hatano et al. demonstrated that raltegravir intensification increased the episomal DNA in a significant percentage of individuals, arguing that HIV replication persists in some subjects despite ART [96]. The same study also reported a decrease in D-dimer levels upon raltegravir intensification, suggesting that residual replication contributes to alterations in the coagulation pathway. As in the Buzon study, the rise in the level of 2-LTR circles in the raltegravir group tended to occur in subjects receiving a PI-based ART regimen. In another raltegravir intensification study, Hatano and colleagues also observed a difference in response to the intensification by the ART regimen, but this time the effect was at the level of RV: although no effect on RV was measured between drug-intensified and placebo groups in the full sample set, limiting the analysis to PI-treated subjects resulted in a higher proportion of subjects with undetectable plasma RNA at week 12 in the raltegravir group [97]. The results of these three intensification trials suggest that residual HIV replication is more likely with PI-containing regimens. Yukl et al. demonstrated that raltegravir intensification did not result in a consistent change in cell-associated HIV RNA levels in blood, duodenum, colon, and rectum [95]. However, in the ileum, where the highest baseline HIV unspliced RNA level and RNA/DNA ratio was measured, the unspliced RNA copy number per 10^6^ CD4+ T-cells decreased in most individuals following raltegravir intensification. Supporting this evidence, they also observed a trend towards an increase in CD4+ T-cell content in the ileum and a decrease in CD8+ T-cell activation in the ileum and blood. 

Although this revealed the ability of raltegravir intensification to perturb the reservoir, supporting the idea that active HIV replication occurs despite ART, most of these studies did not show a significant and sustained decrease in the level of RV. This may indicate that RV stems mostly from the reactivation of reservoir cells and not from ongoing HIV replication. Similarly, Puertas et al. did not find much concordance between HIV sequences in RV and episomal DNA from peripheral blood in three ART-treated individuals, suggesting that RV predominantly originates from stable reservoirs [111].

Intensification studies with other compounds include dolutegravir addition to a three-drug regimen [99], which did not significantly increase the level of 2-LTR circles in peripheral blood. It should be noted that only 12% of participants were treated with PIs in that study, which might explain the absence of a dolutegravir effect. ART intensification with maraviroc (an antagonist of CCR5, the main HIV receptor) has also been undertaken by a number of groups. Upon addition of maraviroc to ART in nine chronically infected individuals, Gutiérrez et al. observed a transient increase in 2-LTR circles and RV, both of which returned to baseline levels by the end of the intensification period [112]. A trend towards a reduction in latently infected cell frequencies was also observed. Contrary to INSTIs, maraviroc was not expected to increase 2-LTR circles, even if it inhibited residual viral replication. Therefore, its effect on 2-LTR circles and RV was explained by possible HIV latency reversal and induction of residual replication by the drug [112,113,114]. However, other groups failed to show any effect of maraviroc on 2-LTR circles or RV [98,115]. Interestingly, the addition of maraviroc to a raltegravir-based ART regimen in individuals starting ART resulted in a faster decline of plasma viremia, as well as lower levels of total HIV DNA and 2-LTR circles in the intensified arm [116], suggesting that suppression of HIV replication by INSTI-based regimens may also be incomplete.

## 5. Differences in Residual Viremia and Other HIV Reservoir Markers between Infected Individuals Treated with Different ART Regimens 

The impact of different ART regimens on HIV persistence has been explored primarily at the level of RV. Historically, most studies compared RV between triple regimens consisting of two NRTIs plus an NNRTI or PI. The majority of these studies reported reduced viremia levels in individuals treated with NNRTI-based ART regimens (Table 1). Remarkably, analysis of the literature survey presented in Table 1 revealed that the studies that reported lower HIV viremia on NNRTI-based as compared to PI-based regimens were significantly larger than studies that reported no difference by regimen (median number of participants 1160 (IQR, 173–1392) vs. 168 (158–334), *p* = 0.041, Mann–Whitney test). Consequently, the studies that did not find a difference by regimen might have lacked sufficient statistical power to do so.

While the design of the studies that compared HIV viremia between ART regimens was either cross-sectional, case-control, or longitudinal, all of them performed comparisons at the level of individual participants, meaning that even longitudinal studies used cumulative measures to compare percentages of individuals with or without RV or low-level viremia. Importantly, most of these longitudinal studies did not account for switches in the drug regimen. To avoid this concern, we recently performed a longitudinal study, including more than 11,000 plasma viral load measurements from 1160 individuals on triple ART with the viral load suppressed to < 20 copies/mL, considering current ART regimens for every measurement individually [139]. While no difference was observed between NNRTI-based and INSTI-based regimens (*p* = 0.18), PI-based treatment was associated with an increased frequency of detectable RV below the limit of quantification as compared to both NNRTI-based (*p* = 0.013) and INSTI-based (*p* < 0.0001) regimens.

These results are in line with the results from intensification studies that suggested that PI-based regimens are less suppressive than other regimens and thus could favor ongoing viral replication in some individuals [94,96,97]. Because PIs have very steep dose-response curves and were shown to block multiple steps in the viral replication cycle, it is possible that relatively small changes in drug concentration can lead to relatively large changes in inhibitory activity [96,140,141,142]. This, coupled to insufficient tissue penetration of a number of antiretroviral drugs, including PIs [71,72,73,74,77,143], can result in suboptimal suppression of residual virus replication in tissue sites by PI-based regimens. An alternative explanation could be the “prescription bias”, as clinicians might preferentially prescribe PI-based regimens to individuals with a worse viro-immunological profile and/or expected poor therapy adherence because PIs impose a relatively high genetic barrier to resistance and consequently will be more “forgivable” to non-adherence. Indeed, first-line PI-based ART regimens were shown to be associated with higher baseline plasma viral loads and lower baseline and nadir CD4+ counts, as well as lower CD4/CD8 ratios [118,120,129,138]. However, numerous studies adjusted for these and other variables (like ART duration) in multivariable models and still reported an independent association of PI-based regimens with RV (Table 1). Moreover, although older PIs were indeed associated with reduced ART adherence [144,145], we and others found no association of current PI-based regimens with the adherence level [132,146]. PI-based regimens were also associated with reduced virological suppression, independently of ART adherence [132,147].

Several groups compared other HIV reservoir markers, such as HIV DNA and cell-associated HIV RNA, in individuals treated with NNRTI-based vs. PI-based ART regimens. The results for cell-associated HIV markers are more controversial than for RV. Nicastri et al. reported lower HIV DNA levels in individuals treated with PI-based regimens, and Sarmati et al. reported no difference by regimen in HIV DNA levels, despite the fact that both studies reported higher RV in PI-treated individuals [118,127]. Kiselinova et al. performed a matched case-control study comparing nevirapine and PIs for RV, total, and episomal HIV DNA and cell-associated HIV RNA (unspliced and multiply spliced) and did not find differences by regimen for any of these markers [131]. It must be noted that Kiselinova et al. matched participants for the duration of PI-based or nevirapine-based regimens, but differences were still observed between the nevirapine- and PI-treated groups in total ART duration and duration of plasma viral load suppression. We recently presented our new unpublished data on the comparison of total HIV DNA and cell-associated HIV unspliced RNA in two independent cohorts of individuals treated with three-drug ART regimens consisting of two NRTIs plus either one NNRTI or one (ritonavir-boosted) PI [148]. No participant matching was performed, but multivariate models were built that were adjusted for age, gender, current and nadir CD4+ count, pre-therapy plasma viremia, duration of virological suppression on ART, NRTI backbone composition, and low-level plasma viremia detectability. In contrast to the aforementioned studies, in both cohorts, the levels of cell-associated HIV RNA and DNA were lower in participants receiving the NNRTI-based compared to the PI-based ART regimens. When stratified by individual NNRTI, unspliced RNA was significantly lower in participants treated with either nevirapine or efavirenz, compared to PI-treated participants, and total HIV DNA was lower in efavirenz-treated than in PI-treated participants. No significant differences in HIV RNA or DNA were observed between the individual NNRTIs or between the individual PIs used. 

As all current antiretroviral drug classes, including NNRTIs and PIs, are not expected to inhibit HIV RNA transcription or virus production, no differences in HIV transcription or virus production by regimen are expected if the drugs are equally potent in suppressing HIV replication (Figure 1). Therefore, the differences in cell-associated HIV RNA levels or RV by ART regimen would suggest that NNRTIs are more potent in suppressing HIV residual replication than PIs, resulting in smaller viral reservoir size. However, recently it has been shown that some NNRTIs, such as rilpivirine, efavirenz, and etravirine, can promote selective apoptosis of infected cells by inducing HIV protease-mediated cytotoxicity [149]. If these NNRTIs are present in cells that are producing viral proteins, they may bind to the reverse transcriptase portion of a newly translated Gag-Pol polyprotein and promote its homodimerization, resulting in premature protease activation. This leads to a decrease in virus production and non-specific cleavage of multiple host proteins, including proteins that induce apoptosis [150,151]. Accordingly, during in vitro HIV latency reversal, the addition of NNRTIs was associated with a large reduction in virus production [152]. Although this mechanism will be nonfunctional in most reservoir cells as only a small proportion of the latter is producing HIV proteins, cells that become reactivated to do so in response to immune stimuli might be selectively killed by this NNRTI action, providing an alternative explanation for the observed lower levels of HIV transcription and virus production in individuals treated with NNRTI-based regimens.

Whether differences in RV or other viral reservoir markers between NNRTI- and PI-treated individuals translate into clinical complications, such as virological failure and development of drug resistance, is also controversial. Riddler et al. conducted a randomized trial comparing efavirenz plus two NRTIs, ritonavir-boosted lopinavir plus two NRTIs, and ritonavir-boosted lopinavir plus efavirenz for the time to virological failure and found that it was significantly longer in the efavirenz group than the lopinavir-ritonavir group [119]. Also, at week 96 of ART, the proportion of participants with <50 copies of plasma HIV RNA per milliliter was significantly higher in the efavirenz group than the lopinavir-ritonavir group. In a systematic review of 48-week efficacy from 15 randomized trials (*n* = 8083), Pozniak et al. determined that participants were significantly more likely to show HIV RNA levels between 50–400 copies/mL while taking first-line boosted PI-based compared with first-line NNRTI-based ART [121]. These results were confirmed by a number of observational studies that showed that PI-based regimens were associated with an increased risk of virological rebound [47,117,124,133,137]. However, Grennan et al. did not find an association of ART regimen and virological failure, although the same study reported a higher number of blips per year in participants on PI-based as compared to NNRTI-based regimens [153]. In contrast, Sungkanuparph et al. did not observe a difference in blip frequency or time to a blip between NNRTI- and PI-treated participants [154].

Some studies reported that INSTI-based regimens are associated with lower RV than other regimens. Pascom et al. performed a very large study that compared cumulative viremia during the first 12 months of ART in 112,243 individuals [155]. INSTI-based regimens were associated with significantly lower cumulative RV compared to both PI-based and NNRTI-based regimens. NNRTI-based regimens were associated with lower RV compared to PI-based regimens, but the level of significance of the latter comparison was not reported. In part, these results reflect faster suppression of plasma viremia after ART initiation by INSTI-based regimens. Lambert-Niclot et al. also reported an association of undetectable (as compared to detectable ultralow) viremia on ART and an INSTI-based regimen [138]. Interestingly, Morón-López et al. recently performed a randomized clinical trial to investigate the impact of switching from a PI-based to a dolutegravir-based regimen on blood and tissue HIV reservoirs and RV [156]. The switch had no impact on HIV reservoir markers in peripheral and ileal CD4+ T cells, but a decrease in RV was observed in the switch group. However, the significance was lost after correction for multiple comparisons. It was concluded that switching might decrease RV in individuals who have a relatively high residual viral load.

Other groups were interested in comparing the ability of various NNRTIs to suppress HIV viremia. Some studies reported that nevirapine has a greater capacity to suppress RV when compared to the widely used NNRTI efavirenz [120,157]. This observation could be explained by better penetration of nevirapine in some anatomical sanctuaries [158]. Interestingly, nevirapine has shown very little in vitro HIV protease-mediated cytotoxicity, while efavirenz was very active [149], arguing that induction of apoptosis of infected cells may not be the major mechanism behind the more pronounced virological suppression by NNRTIs.

## 6. Future Perspectives

Although many studies have been undertaken to better understand the effect of different ART regimens on the persistence of HIV reservoirs on therapy, a number of unanswered questions remain. First, the impact of the different regimens on immune dysfunction, such as immune activation, systemic inflammation, microbial translocation, mitochondrial dysfunction, and oxidative stress, is unclear. It has been suggested that heightened immune activation and inflammation may be both a cause and a consequence of HIV reservoir persistence and residual viral replication, in particular, in tissues [23,159]. Therefore, ART regimens may have a direct and indirect effect on the host immune function. However, data on the influence of different ART regimens on immune activation and inflammation have so far been mixed [160]. In a randomized trial, Hileman et al. observed more pronounced changes in systemic and vascular inflammation and monocyte activation markers after initiating INSTI-based compared with NNRTI-based ART [161]. However, other randomized studies did not find differences in markers of inflammation, immune activation, T-cell senescence, and exhaustion between INSTI-based and PI-based ART regimens [162,163]. Besides, the switch from a PI-based to an INSTI-based regimen did not lead to a change in immune activation or inflammation markers [156]. No clear differences have been found between NNRTI-based and PI-based regimens as well [160]. Second, in the context of HIV curative interventions, it is important to determine the impact of the different regimens on time to viral rebound and viral set point after ART cessation as both of these measures likely correlate with the size of the replication-competent HIV reservoir. Li et al. reported that both NNRTI-based ART regimen and lower levels of cell-associated HIV RNA were associated with a longer time to viral rebound [164]. Our group recently reported that cell-associated HIV RNA was predictive of both time to and magnitude of viral rebound after discontinuation of ART initiated during primary HIV infection [165]. Although ART regimen was not directly associated with time to rebound in that study, our finding that levels of cell-associated HIV RNA and DNA were lower in individuals treated with NNRTI-based regimens [148] argues that further research is needed to determine the influence of ART composition on the clinical measures of the reservoir, such as the time to viral rebound. Third, it will also be important to investigate the effects of different ART regimens on tissue levels of HIV DNA or RNA, especially given the differences in drug penetration into tissues between different regimens.

The existence of ongoing HIV replication on ART remains a highly controversial topic and continues to fuel a longstanding debate in the field. Several reports have suggested that this phenomenon likely occurs in some individuals and that this depends on various factors, including ART regimens. A number of dual therapies recently appeared in the HIV treatment guidelines. Interestingly, although the possibility of ongoing replication despite ART has been highlighted as a potential concern associated with dual therapy [166], the first data recently presented on RV with the dual treatment with lamivudine + dolutegravir did not show any difference with triple therapy at viral load < 40 copies/mL [167].

## Figures and Tables

**Table 1:** Studies that compared low-level viremia and RV on ART between NNRTI- and PI-based triple regimens.

Study (in Chronological Order)	Design	*n* Total	*n* by ART Regimens	ART Regimens Compared	Outcome Measure	Difference NNRTI vs. PI	Ratio Univariable (95% CI)	*p* Uni	Ratio Multivariable (95% CI)	*p* Multi
Palmisano 2005 [51]	Cross-sectional	84	56 (NNRTI), 22 (PI), 6 (NRTI)	Current NNRTI vs. PI	% with RV (> 2.5 copies/mL)	NNRTI < PI		0.048	aOR^i^_NNRTI_ = 0.32 (0.12−0.86)	0.02
				Initial NNRTI vs. PI		NNRTI < PI		0.009	aOR_NNRTI_ = 0.31 (0.13−0.77)	0.01
Maldarelli 2007 [69]	Cross-sectional	158	130 (PI), 28 (NNRTI)	Current NNRTI vs. PI	RV (SCA^ii^)	No difference		0.29		
Geretti 2008 [117]	Longitudinal	1386	539 (NNRTI), 457 (PI), 125 (NRTI), 165 (other)	ART regimens when first achieving pVL^iii^ < 50 copies/mL	% with low-level VL rebound (50–400 copies/mL)	NNRTI < PI	OR^iv^_bPI_ = 1.37 (1.00–1.90), OR_PI_ = 1.70 (1.19–2.43)	0.0005	aOR_bPI_ = 1.39 (1.00−1.94), aOR_PI_ = 1.48 (0.97−2.26)	0.01
					% with virological failure (confirmed pVL > 400 copies/mL)	NNRTI < PI (trend)	OR_bPI_ = 1.98 (1.08–3.62), OR_PI_ = 1.28 (0.69–2.38)	0.06	aOR_bPI_ = 1.88 (1.02−3.46), aOR_PI_ = 1.23 (0.66−2.31)	0.09
Nicastri 2008 [118]	Cross-sectional	319	104 (PI), 166 (NNRTI), 49 (NRTI)	Current ART regimens	RV (LoD^v^ = 2.5 copies/mL)	No difference		0.5		
					% with RV above the median	NNRTI < PI		0.002		
Riddler 2008 [119]	Randomized trial, Longitudinal	753	250 (EFV^vi^-based), 253 (LPV/r^vii^-based), 250 (NRTI-sparing)	ART regimens	time to virological failure	EFV < LPV/r	HR^viii^_EFV_ = 0.63 (0.45–0.87)	0.006		
					% with pVL < 50 copies/mL at 96 weeks ART	EFV < LPV/r		0.003		
Bonora 2009 [120]	Cross-sectional	154	48 (NVP^ix^), 57 (EFV), 49 (LPV/r)	Current ART regimens	% with RV ( > 2.5 copies/mL)	NVP < other drugs			aOR_NVP_ = 0.19−0.82	0.013
						LPV/r > other drugs			aOR_LPV/r_ = 0.91−5.17	0.08
Pozniak 2009 [121]	Systematic review of 15 randomized trials	8083	4475 (NNRTI), 3608 (PI)	First-line NNRTI vs. first-line PI	% with pVL between 50–400 copies/mL at 48 weeks ART	NNRTI < PI		< 0.001		
Charpentier 2012 [122]	Longitudinal	656	321 (PI), 220 (NNRTI), 115 (other)	ART regimens	% with low-level viremia (20–50 copies/mL)	No difference		0.23		
Doyle 2012 [47]	Longitudinal	1247	268 (NNRTI), 492 (PI), 30 (NRTI), 101 (other)	ART regimens	% with pVL between 40–49 copies/mL vs. detectable < 40 copies/mL vs. undetectable	NNRTI < PI		< 0.0001		
					% with viral rebound to > 50 copies/mL within one year	NNRTI < PI	HR_NNRTI_ = 0.27 (0.16–0.45)	< 0.0001	aHR^x^_NNRTI_ = 0.40 (0.21−0.77)	0.002
					% with viral rebound to >400 copies/mL within one year	NNRTI < PI	HR_NNRTI_ = 0.32 (0.14–0.71)	0.007	aHR_NNRTI_ = 0.46 (0.17−1.23)	0.23
Gianotti 2012 [123]	Longitudinal	739	204 (NNRTI), 414 (PI), 46 (NRTI), 75 (other)	ART regimens	% with RV ( > 1 copy/mL)	NNRTI < PI		0.001		
Maggiolo 2012 [124]	Longitudinal	1214	666 (NNRTI), 450 (PI), 98 (other)	Current NNRTI vs. PI	Risk of virological failure	NNRTI < PI		< 0.0001		
					% with RV ( > 3 copies/mL)	NNRTI < PI		< 0.0001		
Martin-Blondel 2012 [125]	Cross-sectional	1392	45% (NNRTI), 43% (PI), 12% (INSTI)	ART regimens	% with pVL between 20–50 copies/mL vs. detectable < 20 copies/mL vs. undetectable	NNRTI < PI		0.0008	aOR_NNRTI_ = 1.45 (1.03−2.04)	0.03
Parisi 2012 [126]	Cross-sectional	180	71 (EFV), 21 (NVP), 83 (PI), 5 (other)	ART regimens	% with pVL 50–1000 copies/mL vs. 21–49 copies/mL vs. 2.5–20 copies/mL vs. < 2.5 copies/mL	No difference		NS^xi^		
Sarmati 2012 [127]	Cross-sectional	420	228 (NNRTI), 192 (PI)	Current NNRTI vs. PI	% without RV ( < 1 copy/mL)	NNRTI < PI	OR_NNRTI_ = 1.73 (1.15–2.60)	0.008		
Zheng 2013 [128]	Cross-sectional	103	65% (NNRTI), 35% (PI)	Current NNRTI vs. PI	RV (SCA)	No difference		NS		
Charpentier 2014 [129]	Cross-sectional	168	60 (EFV), 108 (PI)	ART regimens	Virological outcome at week 48 ART (pVL < 50, < 20, and < 1 copies/mL)	No difference		NS		
Vancoillie 2014 [130]	Longitudinal	173	49 (NNRTI), 122 (PI), 2 (other)	Initial NNRTI vs. PI	% with long-term low-level viremia (20–250 copies/mL) vs. undetectable pVL	NNRTI < PI		0.002	aOR_PI_ = 2.90 (1.20–6.97)	0.017
Kiselinova 2015 [131]	Case-control	161	81 (NVP), 80 (PI)	Current NVP vs. PI	% without RV (SCA)	No difference	OR_NVP_ = 1.53 (0.82–2.86)	0.17		
Konstantopoulos 2015 [132]	Cross-sectional	128	45 (NNRTI), 83 (PI)	Current NNRTI vs. PI	% with low-level viremia (50–1000 copies/mL)	NNRTI < PI	HR_bPI_ = 2.7 (1.1–6.4)	0.03	aHR_bPI_^xii^ = 3.1 (1.3−7.4)	0.01
							HR_PI_ = 3.0 (1.1–6.4)	0.03	aHR_PI_ = 3.1 (1.2–8.3)	0.02
Leierer 2015 [133]	Cross-sectional	2276	1300 (NNRTI- or INSTI), 976 (PI)	Current NNRTI/INSTI vs. PI	% with low-level viremia ( < 200 copies/mL) vs. BLQ^xiii^	NNRTI/INSTI < PI	OR_PI_ = 1.52 (1.15–2.01)	0.003	aOR_PI_ = 1.54 (1.15–2.06)	
					% with virological failure (pVL ≥ 200 copies/mL) vs. BLQ	NNRTI/INSTI < PI	OR_PI_ = 2.78 (1.74–4.42)	< 0.001	aOR_PI_ = 2.36 (1.45–3.83)	
McKinnon 2016 [134]	Longitudinal	356	204 (NNRTI), 152 (PI)	Current NNRTI vs. PI	% with pVL < 40 copies/mL among those with pVL < 200 copies/mL	NNRTI < PI^xiv^	OR_NNRTI_ = 2.26 (1.30–3.94)	0.004	aOR_NNRTI_ = 2.06 (0.99–4.28)	0.05
						NNRTI < PI^xv^	OR_NNRTI_ = 1.98 (1.17–3.37)	0.01	aOR_NNRTI_ = 1.99 (1.14–3.48)	0.02
					% with RV among those with pVL < 40 copies/mL (*n* = 348)	No difference^xiv^	OR_NNRTI_ = 0.77 (0.52–1.14)	0.2	aOR_NNRTI_ = 0.86 (0.56–1.31)	0.5
						NNRTI < PI^xv^	OR_NNRTI_ = 0.53 (0.34–0.85)	0.008	aOR_NNRTI_ = 0.54 (0.34–0.86)	0.01
Riddler 2016 [135]	Cross-sectional	334	61% (NNRTI), 28% (PI), 11% (other)	Initial NNRTI vs. PI	% with RV at 192 and 208 weeks of ART (SCA)	No difference	OR_PI_ = 1.16 (0.79–1.61)	0.45	aOR_PI_ = 1.30 (0.88–1.92)	0.19
Gianotti 2018 [136]	Longitudinal	771	244 (NNRTI), 254 (PI), 234 (INSTI), 39 (other)	First-line NNRTI vs. first-line PI	% of time on ART spent with RV (detectable < 50 copies/mL)	NNRTI < PI		< 0.0001		
Geretti 2019 [137]	Longitudinal	6599	4889 (NNRTI), 1710 (PI)	First-line NNRTI vs. first-line PI	% with virological suppression on ART	NNRTI < PI	HR_PI_ = 0.69		aHR_PI_ = 0.70 (0.65–0.74)	< 0.001
					% with viremia ( > 50 copies/mL)	NNRTI < PI	HR_PI_ = 2.27		aHR_PI_ = 2.17 (1.88–2.51)	< 0.001
Lambert-Niclot 2019 [138]	Longitudinal	717	211 (NNRTI), 419 (PI), 87 (INSTI)	First-line ART regimens	% achieving ultralow VL - not detected on ART	No difference				
					% with virological rebound on ART	NNRTI < PI	HR_NNRTI_ = 0.60 (0.43–0.84)^xvi^	0.003	aHR_NNRTI_ = 0.76 (0.50–1.15)	0.2
							HR_PI_ = 1.20 (0.88–1.64)^xvi^	0.2	aHRPI = 1.00 (0.69–1.43)	0.9
Darcis 2020 [139]	Longitudinal	1160	Samples: 4210 (NNRTI), 3280 (PI), 3555 (INSTI)	Current NNRTI vs. PI	% samples with detectable RV (all samples < 20 copies/mL)	NNRTI < PI			aOR_NNRTI_ = 0.85 (0.74–0.97)	0.013

^i^ aOR, adjusted odds ratio. ^ii^ SCA, single-copy assay. ^iii^ pVL, plasma viral load. ^iv^ OR, odds ratio. ^v^ LoD, limit of detection. ^vi^ EFV, efavirenz. ^vii^ LPV/r, ritonavir-boosted lopinavir. ^viii^ HR, hazard ratio. ^ix^ NVP, nevirapine. ^x^ aHR, adjusted hazard ratio. ^xi^ NS, not significant. ^xii^ bPI, boosted protease inhibitor. ^xiii^ BLQ, below limit of quantification. ^xiv^ ART initiated 1996–2001. ^xv^ ART initiated 2002–2009. ^xvi^ Compared to all regimens.
